# Our emergency surgical approach in a case with migration of the atrial septal defect occluder device into left atrium and displacement on mitral valve

**DOI:** 10.1186/1749-8090-8-S1-P175

**Published:** 2013-09-11

**Authors:** U Yetkin, K Ergunes, V Ermen, O Ergene, I Yurekli, A Gurbuz

**Affiliations:** 1 Department of Cardiovascular Surgery, Izmir Katip Celebi University Ataturk Training and Research Hospital, Izmir, Turkey; 2 Department of Cardiology, Izmir Katip Celebi University Ataturk Training and Research Hospital, Department of Cardiology, Izmir, Turkey

## Background

Closure of secundum atrial septal defects (ASD) via transcatheter technique provides prominent symptomatic recovery and the diameters of cardiac chambers shrink definitely.

## Methods

Our case was a 47-year-old male. After diagnosis of secundum type ASD, he underwent transcatheter closure of the defect with 26 mm Amplatzer septal occlude device. But migration of the ocluder device occurred and an emergency surgery was planned due to this complication.

## Results

After median sternotomy and vertical incision of the pericardium, standard aortic and bicaval venous cannulation was carried out. Following the cross-clamping of the aorta antegrade blood cardioplegia was introduced. Right atriotomy was done. ASD occluder device was detected within the left atrium settled on the mitral valve which was observed through 50x25 mm fossa ovalis type secundum ASD. After transseptal removal of the device from the left atrium, the defect was repaired using a polytetrafluoroethylene cardiac patch. His intensive care unit stay was 2 days and he was discharged from the hospital on 7th postoperative day. His 1st, 3rd and 6th follow-up visits were free of events and repeated echocardiographic examinations revealed no residual shunting.

## Conclusions

One must keep in mind that transcatheter closure has such complications as migration of the device and development of residual shunting. In case of occurrence of such complications, the problem may be solved by standard open cardiac surgery under cardiopulmonary bypass.

